# Latin America: ripe for cutting-edge research proposals for prevention and control of *Helicobacter pylori*

**DOI:** 10.1007/s10552-012-0129-1

**Published:** 2013-01-10

**Authors:** Eduardo Lazcano-Ponce, María Elena Martínez

**Affiliations:** 1 Centro de Investigación en Salud Poblacional, Instituto Nacional de Salud Pública, Universidad No. 655 Col. Santa María Ahuacatitlán, Cerrada Los Pinos y Caminera, C.P. 62100 Cuernavaca, Morelos Mexico; 2 Moores UCSD Cancer Center, Room 3023, 3855 Health Sciences Drive, #0901, University of California, La Jolla, San Diego, CA 92093-0901 USA

One of the greatest areas of scientific progress has been the identification of certain infectious agents that are implicated in the carcinogenic process. This new knowledge has had broad repercussions not only in the application of new primary prevention options, such as prophylactic vaccines, but also in the definition of infection biomarkers that can be used as screening alternatives or as prognostic markers for neoplasia. We now know that chronic infection with the pathogenic bacteria *Helicobacter pylori* (*H. pylori*) is associated with gastrointestinal tract disorders, ranging from chronic gastritis to gastric adenocarcinoma; there is also evidence of a possible association with gastric lymphoma and peptic ulcer. Published reports support the existence of geographic regions where this bacterium is endemic and where *H. pylori* colonization occurs at a very early age in populations living in precarious sanitary conditions. For this reason, gastric cancer is one of the most common cancers in the world in low-resource countries and, as noted in the accompanying articles [1, 2], one with great heterogeneity in terms of incidence and mortality in Latin America. As a result, this geographic region constitutes a fascinating setting for research and provides unique and important opportunities for gastric cancer prevention.

From a public health perspective, there are currently two major lines of research and action in regard to *H. pylori* and gastric cancer. On the one hand, we should gain a more precise understanding of the natural history of *H. pylori* infection and, as a consequence, learn about the triggering mechanism(s) related to chronic exposure in the development of gastric adenocarcinoma. However, we also know that reducing future incidence of gastric cancer is possible by improving the living conditions of the general population from a hygienic perspective, as confirmed by Porras et al. [3] in this special edition. Evidence now exists, which established *H. pylori* as a part of normal human microbiota [4]. This line of work indicates that this bacterium has a special tropism for gastric mucosa, that strains with toxic potential exist, and that the bacteria secrete proteins such as vacA [5], which produce vacuolization in cultures of human epithelial cells as well as increased capacity to modify molecules. Given the existing evidence from biomedical and epidemiological research underscoring the high degree of genetic diversity in *H. pylori* [6], prevention is arguably very complex.

Research groups in Latin America have generated an invaluable scientific tradition for generation of new hypotheses [7], evidence for interventions [8], and high-impact publications [9] related to the study of the carcinogenic process in gastroduodenal mucosa. Recently, a Latin American *H. pylori* research consortium was formed with the primary mission of evaluating effectiveness, feasibility, and cost of eradication using an antimicrobial regimen specifically for Latin America. Results are encouraging and demonstrate the technical and academic abilities of the participating groups [10]. They also indicate that treatment regimens for successful eradication need to be region-specific; thus, adopting successful treatment regimens from high-income countries may not be the best solution for lower-income countries. Adding to this line of work, results of a diverse set of research studies conducted in various regions of Latin America are presented in this special issue, which reveal the great heterogeneity that exists in the region. These publications include topics such as potential solutions for gastric cancer surveillance in low-resource countries [11], ecological data in support of the altitude–gastric cancer association [1], assessment of the utility of serologic biomarkers for risk of gastric cancer in three Latin American countries [2], confirmation of risk factors for *H. pylori* infection [3], and a meta-analysis of a variety of risk factors and gastric cancer risk [12].

The work presented in this special edition of the journal along with the recently formed consortium underscores the enormous potential that multi-centric regional studies have for addressing the magnitude of health problems, and even more important, identify solutions for prevention of future disease. These invaluable resources allow us to postulate new research hypotheses and to consolidate a regional group, which can contribute to improving therapeutic strategies for prevention and control of gastric cancer and build the foundation for evidence-based regional public policies aimed at improve the quality of life of the population.
